# Skeletal Development and Deformities in Tench (*Tinca tinca*): From Basic knowledge to Regular Monitoring Procedure

**DOI:** 10.3390/ani11030621

**Published:** 2021-02-26

**Authors:** Ignacio Fernández, Francisco Javier Toledo-Solís, Cristina Tomás-Almenar, Ana M. Larrán, Pedro Cárdaba, Luis Miguel Laguna, María Sanz Galán, José Antonio Mateo

**Affiliations:** 1 Aquaculture Research Center, Agro-Technological Institute of Castilla y León (ITACyL), Ctra. Arévalo, 40196 Zamarramala, Segovia, Spain; critoal@gmail.com (C.T.-A.); ita-largaran@itacyl.es (A.M.L.); pcardaba@gmail.com (P.C.); lagcarlu@itacyl.es (L.M.L.); 2 Department Biology and Geology, Ceimar-University of Almería, 04120 Almería, Spain; fj.toledos@gmail.com; 3 Consejo Nacional de Ciencia y Tecnología (CONACYT), Av. Insurgentes Sur 1582, Alcaldía Benito Juárez, Mexico City C.P. 03940, Mexico; 4 Centro Veterinario tus Mascotas, c/ Real n° 19, 40194 Trescasas, Segovia, Spain; tusmascotascv@gmail.com; 5 Tencas Mateo S.L., C/ Campo, 17, 40297 Sanchonuño, Segovia, Spain; tencastencas@gmail.com

**Keywords:** fish quality, deformities, bone, cartilage, double staining, X-ray analysis

## Abstract

**Simple Summary:**

Fish skeletal development and incidence of skeletal deformities are important factors to warrant aquaculture success. Skeletal deformities reduce fish viability, growth, and feed efficiency but also degrade the consumer’s perception of aquaculture products. Some skeletal deformities would also decrease animal wellbeing. Tench (*Tinca tinca*) is a freshwater species cultured in ponds, highly demanded in particular regions of Europe and a promising species for aquaculture diversification. Determining the onset of the different skeletal structures may help fish farmers to adapt and improve rearing practices (e.g., water temperature, feeds composition, etc.) to decrease the incidence of skeletal deformities. At the same time, monitoring the incidence of skeletal deformities represents a valuable decision-making tool to improve efficient use of facilities and resources.

**Abstract:**

Skeletal deformities reduce fish viability, growth, wellbeing, and feed efficiency but also degrade the consumer’s perception of aquaculture products. Herein, the skeletal development and the incidence of skeletal deformities in tench (*Tinca tinca*) reared in semi-extensive conditions has been described in detail for the first time. Larval skeletons were assessed through an acid-free double-staining procedure in 157 individuals, while 274 specimens at the juvenile stage were evaluated through X-ray analysis. The first skeletal structures to be formed were those related with breathing and feeding activities (e.g., Meckel’s cartilage and opercula) and were visible in larvae of 4 mm of standard length (SL). The axial skeleton was fully ossified in larvae of 12–17 mm of SL, and the caudal fin complex in larvae with 17–26 mm of SL. At the larval stage, no upper-jaw or opercula deformities were observed, while a low incidence (1–9%) of other severe deformities in the heads of the fish (e.g., lower-jaw deformities) were reported. The incidence of vertebral deformities in tench reared in natural ponds was considerable in larvae (54%) and juveniles (52%). Vertebral deformities (fusion and compression) were the most common deformities found in tench larvae (approximately 30%) and vertebral shape deformity in juveniles (around 10%), being mainly located in the caudal region. Thus, a regular monitoring of the skeletal deformities in tench might help to identify better rearing protocols and improve product quality sold at markets. Characterizing the skeletal development not only in semi-extensive systems such as artificial and natural ponds but also under intensive rearing conditions, seems vital for a sustainable and profitable European tench aquaculture.

## 1. Introduction

Tench (*Tinca tinca* Linneo, 1758) is a freshwater epibenthic cyprinid hypothesized to be native in most of European countries. Typically, tench inhabit shallow, densely vegetated lakes and backwaters. Nowadays, although widespread and considered as a least-concern species by the International Union for Conservation of Nature (IUCN), they are locally threatened by river engineering and wetlands degradation, particularly the spawning areas, with significant decreases in population numbers [1].

Tench have been cultured in ponds from the XVI century. In Eastern European countries, it is reared in ponds and sold at a weight of over 500 g. In particular regions of Southern European countries (Spain and Italy), it is a highly demanded and valued (between 16–18 € kg^−1^) gastronomic product at lower size (80–120 g), being traditionally reared in extensive monoculture natural ponds [2]. Tench have been also produced for natural restocking, for angling, and as ornamental fish in different aquatic environments [3,4]. However, European production has decreased from more than 5000 to 2400 tons during the last decade, with France, Russia, Poland, Germany, the Czech Republic, and Spain being the major producers [5]. In Spain, production has been reduced, hypothetically due to a significant reduction of wetlands by successive dry events, the lowering of water quality in natural ponds, and/or the constant loss of population in rural areas. Indeed, several uncontrollable factors in ponds can influence fish survival, growth, and reproduction (e.g., environmental temperature). Therefore, implementing regular monitoring procedures as decision-making tools for fish farmers is urgently required to assess whether a rearing protocol or a fish batch should be optimized or discarded, respectively. 

Tench might be a promising species for aquaculture diversification due to several physiological and biochemical features. Tench clearly shows a sexual dimorphism, pelvic rays being more robust, longer, and extending beyond the anus in males. It is tolerant to low oxygen concentrations and salinities (up to 12%). It normally feeds on detritus, benthic animals and plant material, so it might be a good candidate for modern aquaculture, accepting low fish oil and fish meal contents on their diets. Furthermore, tench can tolerate a wide range of environmental temperature (from 10 to 29 °C) and can be easily handled during husbandry procedures (such as classification and/or hormonal injection), because specimens can resist to air exposure over quite large periods (minutes). From a nutritional and biochemical point of view, tench meat has a fine flavor, with indices and rations related to human health (atherogenic index, thrombogenic index, PUFA/SFA, and n-6/n-3 ratios) in accordance with values recommended for high-quality food for humans [2].

Some key knowledge on their biology and husbandry practices has been obtained since the 1980s. Hormonal injection and larval rearing protocols have been developed, including eggs desticking procedure and incubation in Zug bottles or Weiss jars of 2–10 L [6,7,8]. Feeding strategy during early larval development initially consisted of providing planktonic crustaceans and rotifers followed by cladocera, copepod, water mites, and chironomids larvae from natural ponds [9]. Currently, *Artemia* is the most common indoor feeding practice, although recent advances at laboratory scale have been done for the development of inert diets [10,11]. Nevertheless, inert diets already proved to be unsuitable, inducing high mortality and deformity rates [2]. As a result, tench aquaculture is still based on extensive or semi-extensive rearing conditions in ponds. In both systems, it is difficult to control environmental parameters, leading to unpredictable productions with high mortality, slow growth, and low quality. Skeletal deformities are known to induce reduced fish welfare, growth, feed-conversion ratio, and quality images if sold in the markets but also increasing mortality [12]. Knowing the specific sequence of skeletogenic events along larval development and the time of appearance of each type and frequency of deformities might represent a key step toward the implementation of regular monitoring at tench hatcheries and nurseries. In this sense, the implementation of regular skeletal quality assessment might allow to optimize rearing conditions, feeding strategies, and high-quality inert diets. Furthermore, increased quality (e.g., reduced incidence of deformities) will also promote good survival when specimens are restocked in natural environments, leading to a more successful conservation of natural fish populations. To date, a wide range of incidence of skeletal deformities through visual inspection of the external morphology have been reported (from 0 to 96.4%) [13].

Herein, two common procedures to assess the incidence of skeletal deformities in farmed fish, the double-staining (alcian-blue–alizarin-red S) protocol and the X-ray analysis were applied in two cohorts of tench from a Spanish company (Tencas Mateo S.L.), representative of the semi-extensive production in natural ponds. Firstly, the double-staining protocol, previously developed for other aquaculture species, was adapted to describe the skeletal development of tench larvae. Secondly, while the adapted protocol was used to determine the onset and incidence of skeletal deformities in larval stages and early juveniles, an X-ray analysis was performed in outgrowing specimens. The present work not only describes the skeletal development of tench for the first time but also recommends the implementation of regular monitoring of skeletogenesis at two different developmental stages as an important decision-making tool to improve efficient use of facilities and resources.

## 2. Materials and Methods

### 2.1. Ethical Statement

All experiments complied with the ARRIVE guidelines [14] and were performed according to 2010/63/EU of the European Parliament and Council, guideline 86/609/EU of the European Union Council and Spanish legislation (RD53/2013) for animal experimentation and welfare. All the persons involved in the experiments have a FELASA class C permit for animal experimentation. The Center for Aquaculture Research has been registered and authorized as a research institution to perform animal experimentation (REGA number: ES401940000293), and an authorization has been already obtained (Ref. n°: 2019/43/CEEA) from the Bioethical Committee of ITACyL in order to fulfill the administrative requirements prior to conducting the planned research.

### 2.2. Lagoons, Rearing Conditions, and Fish Sampling

Fish broodstock were collected from natural lagoons of Tencas Mateo S.L., maintained in a freshwater recirculation system, and induced to spawn with hormonal manipulation. Females and males were separated and maintained at 24 °C under natural photoperiod and temperature conditions with densities of less than 2 kg m^−2^. Artificial fertilization, eggs incubation, and hatching were performed based on protocols previously published [4,6,8,15]. When females showed external signs of advanced gonadal development, induction of spawning was done with intramuscular injections of luteinizing hormone-releasing hormone (0.02 mg Kg^−1^ body weight; Sigma-Aldrich L4513, Madrid, Spain). Males did not receive any hormonal treatment. After 30 h post-injection, females and males were stripped, and gametes were collected and fertilized. Fertilized eggs were subjected to a desticking procedure using Alcalase® (Sigma-Aldrich P4860, Madrid, Spain) and incubated at 22 °C for 2 days in Zug bottles. Hatched larvae were released and reared in natural ponds (approximately 150 m^−2^). Larvae were fed on natural zooplankton, including daphnia, rotifers, and copepods among others, for 4 months within the natural ponds of Tencas Mateo S.L. Along this period, water temperature ranged from 16 °C to 26 °C, and dissolved oxygen between 4.0 to 8.5 mg L^−1^. Outgrowing phase was done in the same type of ponds where, in addition to the naturally inhabiting zooplankton, commercial feed was regularly supplied.

In order to describe the skeletogenesis and incidence of deformities in tench larvae and early juveniles, specimens were sampled at 5, 7, 30, 42, 65, and 85 days postfertilization (dpf), and a total of 157 specimens (>15 per sampling time) was evaluated. Additionally, an X-ray analysis of 247 1-year-old specimens was also performed in order to explore its suitability for the identification of deformed juveniles at later stages of development.

### 2.3. Growth and Skeletal Development

At each sampling time, collected specimens were sacrificed with an overdose of MS-222 (Sigma-Aldrich, E10505, Madrid, Spain). Its standard length (SL) was measured, and larvae were rinsed in distilled water and fixed in 4% buffered (pH 7.4) formaldehyde either for 2 hours at room temperature (larvae with SL < 1 cm) or at 4 °C for 24 h (larvae/juveniles with SL > 1 cm). Afterwards, specimens were gradually dehydrated in an increasing series of alcohol (EtOH) solutions (25%, 50%, 75%, and 100%). The skeletal double-staining protocol was performed with an adapted protocol from [16]. Briefly, EtOH solution was removed and larvae were immersed in a cartilage staining (0.02% Alcian blue 80 mM MgCl_2_; Sigma-Aldrich A3157 and M8266, Madrid, Spain) solution for 8 h, washed with distilled water, and immersed in a bone-staining (0.005% alizarin red S; Sigma-Aldrich A5533, Madrid, Spain) solution for 1–2 h. Larvae were washed in distilled water and bleached in a 1.5% hydrogen peroxide and 1% hydroxide potassium solution for 1–2 h accordingly to larvae size. Every 5 to 10 minutes clearance of specimens was checked. Once the bleaching solution did not remove more pigments and/or overstaining from fish larvae/juvenile, specimens were transferred to a 1% hydroxide potassium solution. At this stage, in specimens larger than 2 cm SL, scales were gently removed with a toothbrush and/or forceps in order to allow a clear visualization of the axial skeleton. Specimens were maintained (for up to several days) within a 1% hydroxide potassium solution until 80% of the natural pigmentation was removed. In fish with > 2 cm SL, an extra bone-staining procedure for 1 h was performed in order to promote a clear staining of the axial skeleton, being the overstaining afterwards removed with 1% hydroxide potassium solution. Finally, specimens were gradually (25%, 50%, and 75% solution series) transferred to a solution of 100% glycerol, where they were stored until skeletal analysis.

To evaluate the degree of mineralization of the skeleton and to identify and quantify the incidence of skeletal deformities, specimens were placed under a Nikon’s SMZ-1500 zoom stereomicroscope. The skeletal elements that appeared at each specimen were counted. Furthermore, their degree of mineralization was evaluated as in the cartilaginous stage, on its onset of ossification and/or with an advanced ossification (when almost all the structure was ossified). Types and incidence of deformities were also identified, particularly as previously defined in [12]. Nomenclature of skeletal structures was adapted from zebrafish (*Danio rerio*), another fish species belonging to *Cyprinidae* family [17,18].

### 2.4. X-ray Analysis

In total, 274 1-year-old specimens were collected, shipped, and stocked at the ITACyL facilities in 500 L tanks. After acclimation, specimens (ranging 7.7 cm to 13.8 cm of SL and 9.2 to 59.5 g of wet body weight) were subjected to X-ray analysis at the facilities of the Centro Veterinario tus Mascotas (Trescasas, Spain). Fish were slightly anaesthetized with MS-222 (80–160 mg/L, depending the fish size), placed on the right side and X-rayed with an EcoRay ULTRA-100 Toshiba® apparatus (CVM Diagnóstico Veterinario SL, Tudela, Spain), under an exposure of 44–45 kv and 15–16 mAs, depending on the fish size. X-ray images were observed with Metron software® (CVM Diagnóstico Veterinario SL, Tudela, Spain). Vertebral deformities were identified, and their classification was adapted from [19]. In this sense, four different categories of vertebral deformities were considered: fusion, compression, elongation, and vertebral shape deformity as any other alteration of the shape of the vertebra other than the three previously enumerated.

## 3. Results

### 3.1. Skeletal Development

**Figure 1:**
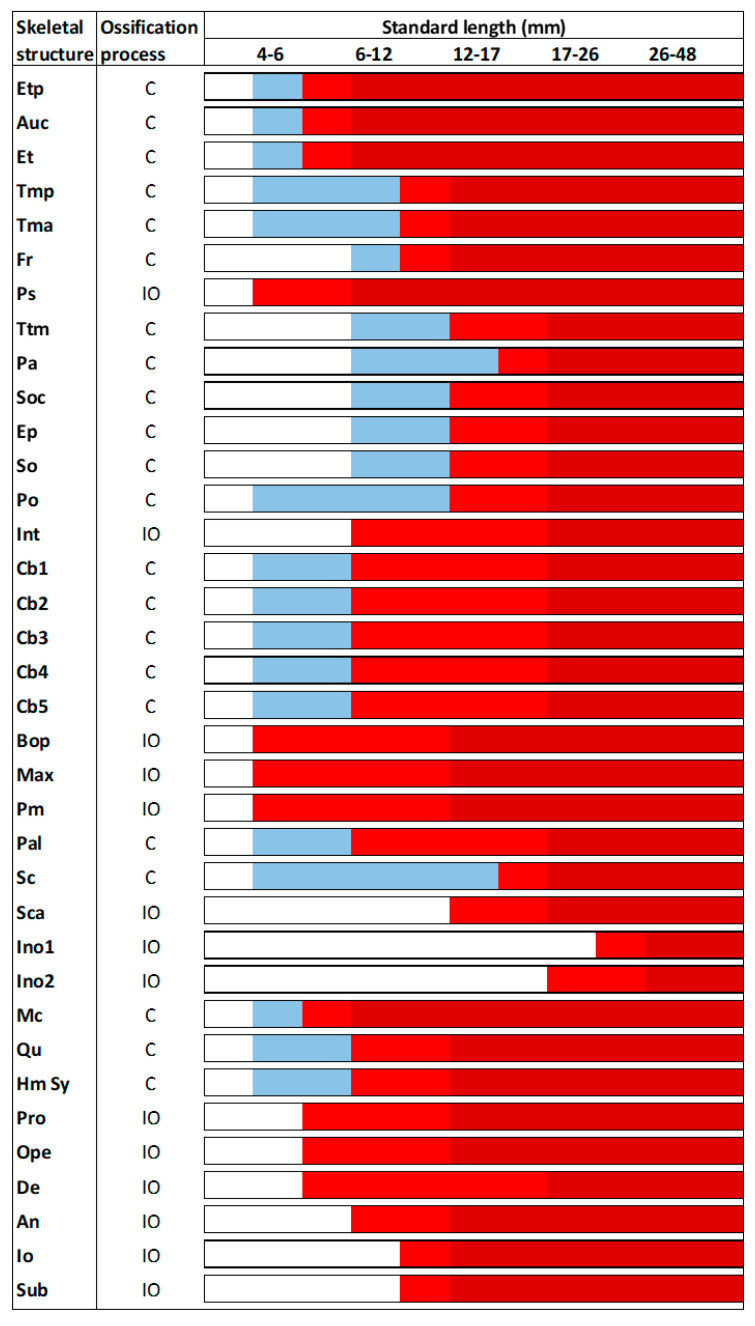
Skeletal formation of main structures of the cranium in tench (*Tinca tinca*) during larval development when reared in natural ponds. Color lines: *white*, structure is still not formed; *blue*, structure is in cartilage stage; *light red*, structure is slightly mineralized; *dark red*, structure is fully mineralized; *C*, chondral ossification; and *IO*, intramembranous ossification. Skeletal structures: *An*, angular; *Auc*, auditory capsule; *Bop*, basioccipital; *Cb1*, ceratobranchial 1; *Cb2*, ceratobranchial 2; *Cb3*, ceratobranchial 3; *Cb4*, ceratobranchial 4; *Cb5*, ceratobranchial 5; *De*, dentary; *Ep*, epiotic; *Et*, epiphyseal tectum: *Etp*, ethmoid plate; *Fr*, frontal; *Hm Sy*, hyosympletic cartilage; *Ino1*, infraorbital 1; *Ino2*, infraorbital 2; *Int*, intercalary; *Io*, inter-opercula; *Max*, maxilla; *Mc*, Meckel’s cartilage; *Ope*, opercula; *Pa*, parietal; *Pal*, palatine; *Pm*, premaxilla; *Po*, prootic; *Pro*, pro-opercula; *Ps*, parasphenoid; *Qu*, quadrate; *Sc*, sclerotic; *Sca*, anterior sclerotic; *So*, sphenotic; *Soc*, supraoccipital; *Sub*, subopercle; *Tma*, taeniamarginalis anterior; *Tmp*, taenia marginalis posterior; and *Ttm*, taenia tecti medialis. Nomenclature adapted from [17,18].

Standard length (SL) growth of tench larvae (from 5 to 85 dpf) followed an exponential curve (y = 0e^0.025x^ with R^2^ = 0.8793) in natural ponds (Supplementary Figure S1). The first skeletal structures to appear were those located in the cranium (Figure 1). The parasphenoid (Ps), basioccipital (Bop), maxilla (Max), and premaxilla (Pm) were visible at tench larvae of 4 mm of SL, and the dentary (De), pro-opercula (Pro) and opercula (Ope) were immediately observable afterwards. All of them appeared as intramembranously ossified structures. At 4 mm SL, tench larvae also presented other skeletal structures at the cranium but in a cartilaginous stage. The Meckel’s cartilage (Mc), auditory capsule (Auc), ethmoid plate (Etp), taenia marginalis posterior (Tmp), taenia tecti medialis (Ttm), ceratobranchials 1–5 (Cb1–5), palatine (Pal), sclerotic (Sc), quadrate (Qu), and the hyosympletic cartilage (Hm Sy) were already visible in larvae ranging 4 to 6 mm of SL. All those structures followed a quite rapid chondral ossification, being already slightly ossified in larvae of 6–12 mm of SL. In general, most cranial structures underwent chondral ossification and most of them were completely ossified in larvae of 17–26 mm of SL, with the exception of the infraorbitals (Ino), which appeared to underwent intramembranous ossification at this size but were only fully ossified in larvae of 26–48 mm of SL.

**Figure 2:**
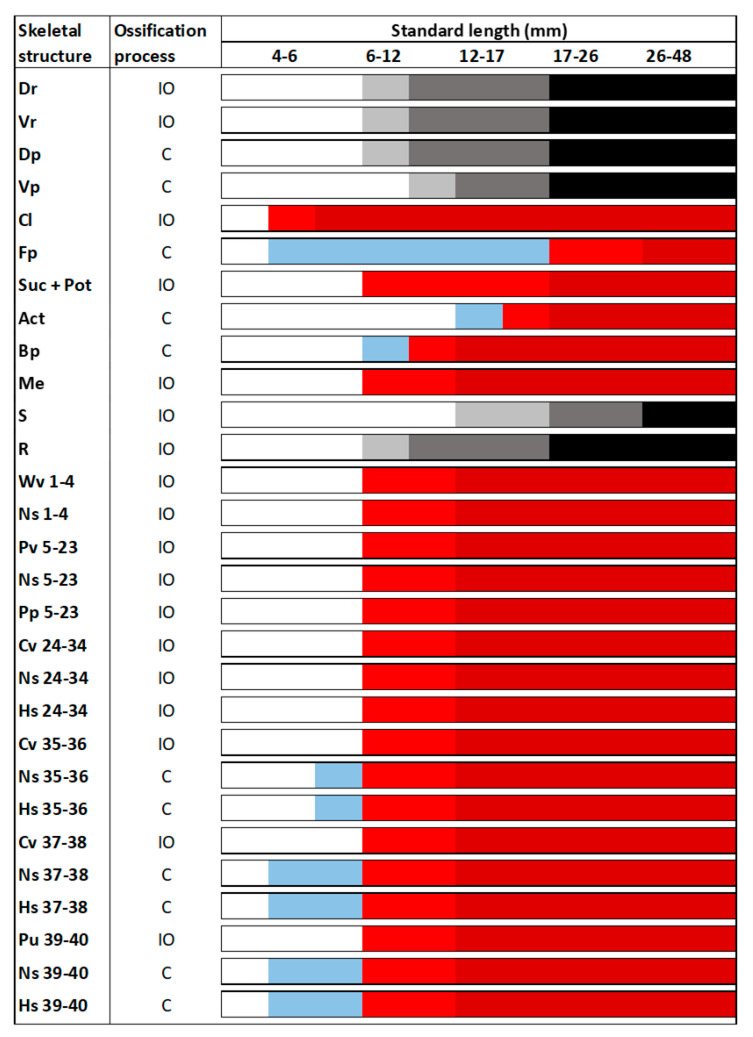
Skeletal formation of main structures of the trunk in tench (*Tinca tinca*) during larval development when reared in natural ponds. Color lines: *white*, structure is still not formed; *blue*, structure is in cartilage stage; *light red*, structure is slightly mineralized; *dark red*, structure is fully mineralized; *light grey*, first elements are appearing; *dark grey*, half of the elements are formed; *black*, all the elements composing this structure are formed and mineralized; *C*, chondral ossification; and *IO*, intramembranous ossification. Skeletal structures: *Act*, actinost; *Bp*, Basipterygium; *Cl*, cleithrum; *Cv*, caudal vertebrae; *Dp*, dorsal pterygiophores; *Dr*, dorsal rays; *Fp*, cartilaginous fin plate; *Hs*, hemal spine; *Me*, metapterygium; *Ns*, neural spine; *Pp*, parapophysis; *Pv*, prehemal vertebrae; *Pu*, preural vertebrae; *Suc + Pot*, supracleithrum and posttemporal structures; *R*, soft rays; *S*, spine; *Vp*, ventral pterygiophores; *Vr*, ventral rays; and *Wv*, Weberian vertebrae. Nomenclature adapted from [17,18].

The axial skeleton also starts to be developed in larvae ranging 4–6 mm of SL (Figure 2). In this case, cleithrum (Cl) was the unique structure undergoing intramembranous ossification already being formed at 4 mm of SL, while cartilaginous structures present at this size were the fin plate (Fp) and the neural (Ns) and hemal (Hs) spines of vertebra 37–40. In contrast to those spines, the other spines of the vertebral column as well as the centra were formed through intramembranous ossification, being the Weberian 3–4 vertebrae the first to be clearly ossified in larvae of 6–12 mm of SL. Vertebral ossification sequence proceeded bidirectionally, toward the head and the urostyle. At the same time, the appendicular structures included in the pectoral, dorsal, pelvic, and anal fins such as the basipterygium (Bp), dorsal (Dp) and ventral (Vp) pterigiophores, dorsal (Dr) and ventral (Vr) rays, and the metapterygium (Me) were developed. Indeed, both the soft rays (R) and the spine (S) were the last ones to be observed in larvae of 12–17 mm of SL.

**Figure 3:**
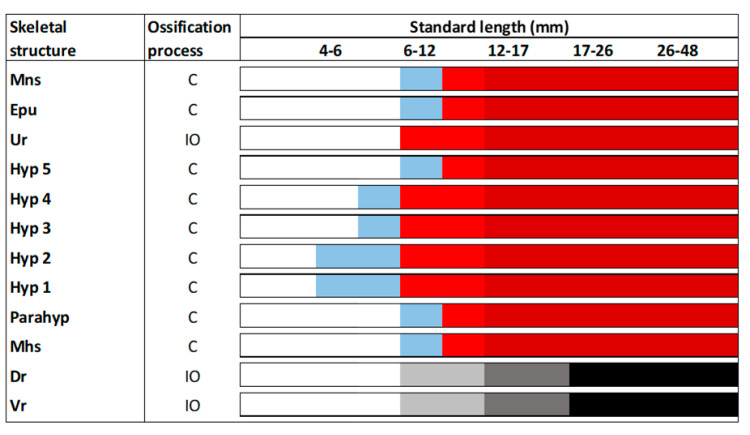
Skeletal formation of main structures of the caudal fin complex in tench (*Tinca tinca*) during larval development when reared in natural ponds. Color lines: *white*, structure is still not formed; *blue*, structure is in cartilage stage; *light red*, structure is slightly mineralized; *dark red*, structure is fully mineralized; *light grey*, first elements are appearing; *dark grey*, half of the elements are formed; *black*, all the elements composing this structure are formed and mineralized; *C*, chondral ossification; and *IO*, intramembranous ossification. Skeletal structures: *Mns*, modified neural spine; *Epu*, epural; *Ur*, urostyle; *Hyp 1-5*, hypurals 1-5; *Parahyp*, parahypural; *Mhs*, modified hemal spine; *Dr*, dorsal rays; and *Vr*, ventral rays. Nomenclature adapted from [17,18].

**Figure 4:**
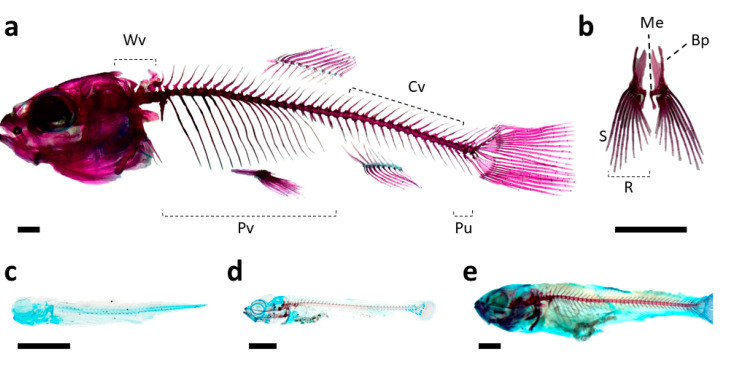
Schematic representation of the skeleton of tench (*Tinca tinca*), particularly of the vertebrae included in the axial skeleton (**a**) and the elements of the pelvic fins (**b**); and representative images of how the ossification proceeds along the larval development: larvae of 3.5 (**c**), 7.0 (**d**), and 12 mm of SL (**e**). Skeletal structures: *Bp*, basipterygium; *Cv*, caudal vertebrae; *Me*, metapterygium; *Pv*, prehemal vertebrae; *Pu*, preural vertebrae; *R*, soft rays; *S*, spine; and *Wv*, Weberian vertebrae. Nomenclature adapted from [17,18]. Scale bar = 1 mm.

In the caudal fin complex, although some elements started to appear at 4–6 mm of SL such as the hypurals (Hyp) 1 and 2, the onset of their ossification begins at 6–12 mm (Figure 3). All the structures composing the caudal fin complex underwent chondral ossification, with the exception of the urostyle (Ur) and the dorsal and ventral rays, which are formed by intramembranous ossification. In this case, the onset of ossification of the urostyle was observed in larvae with 6–12 mm of SL, while the ossification of the rays proceeded sequentially, becoming fully developed at 17–26 mm of SL. As a reference material for future studies, a schematic representation of the different skeletal structures composing the skeleton of the tench, as well as some representative figures of how the ossification proceeds along larval development, is provided in the Figure 4.

### 3.2. Larval Skeletal Deformities

**Figure 5:**
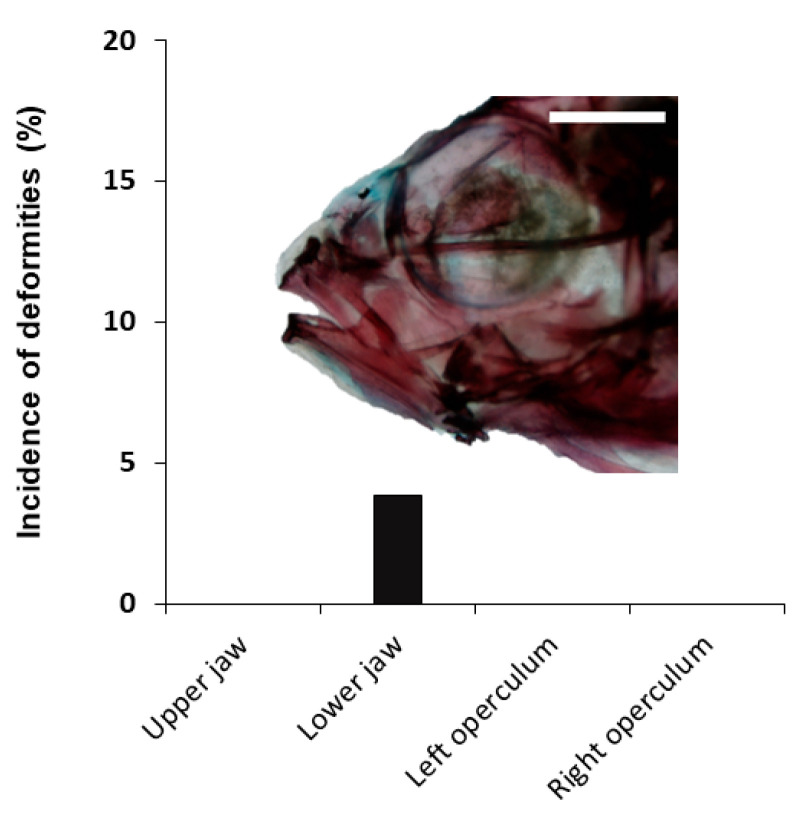
Incidence of skeletal deformities in the cranium of tench (*Tinca tinca*) during larval development when reared in natural ponds through double-staining analysis. Embedded image shows a lower-jaw (short) deformity. Scale bar = 1 mm.

**Figure 6:**
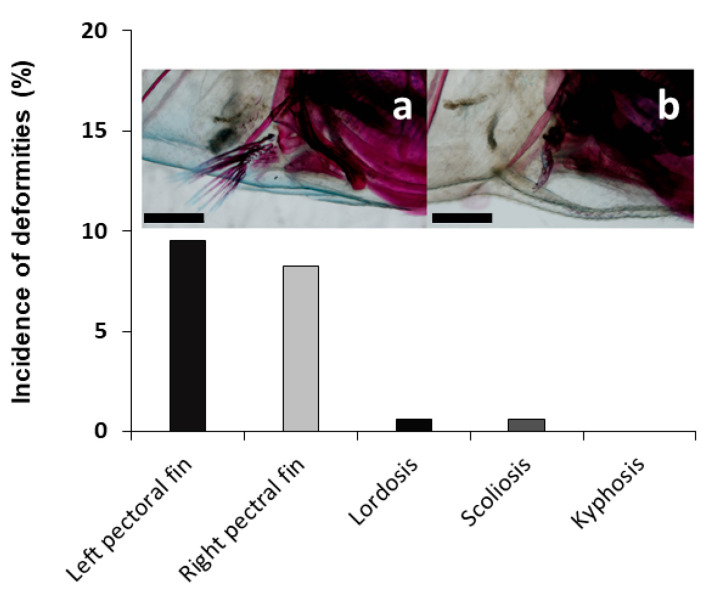
Incidence of skeletal deformities in the pectoral fins and abnormal curvatures of the axial skeleton in tench (*Tinca tinca*) during larval development when reared in natural ponds through double-staining analysis. Embedded images show a normal specimen (**a**) and a specimen with no right pectoral fin (**b**). Scale bar = 1 mm.

Among the 157 individuals analyzed during larval development, 54.1% showed at least one skeletal deformity. In the cranial region (Figure 5), a low incidence (3.82%) of lower-jaw (short) deformity was found among the different skeletal deformities identified. Other severe deformities in the cranial region like upper-jaw and opercular deformities were not identified. Similarly, some severe deformities were also observed in the trunk region (Figure 6). Some individuals showed scoliosis and lordosis, although both were reported with a very low incidence (0.64%), but none of them showed kyphosis. Specimens showing absence of one of the pectoral fins (partial or total absence) were more frequently identified, with 9.55 and 8.28% of the specimens lacking the left or right pectoral fin, respectively.

**Figure 7:**
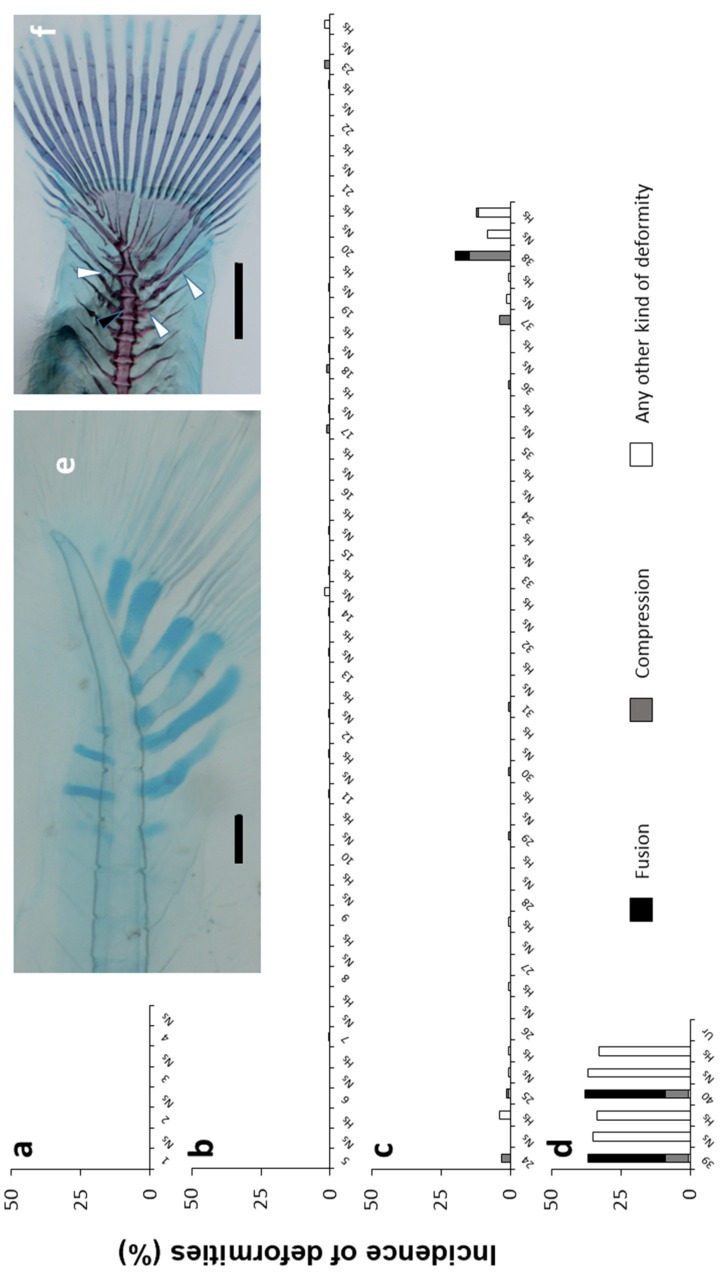
Incidence of deformities along the axial skeleton: Weberian vertebrae (**a**); prehemal vertebrae (**b**); caudal vertebrae (**c**); and preural vertebrae (**d**). Types of deformities represented: fusion (F), elongation (E), compression (C), or any other kind of deformity (D). Nomenclature adapted from [19]. Embedded images: (**e**) detail of growing hypurals, first neural and hemal spines at cartilage stage in a tench larvae, scale bar = 0.1 mm; (**f**) detail of a fully mineralized caudal fin with a vertebral fusion (black arrowhead) and misplaced neural and hemal spines (white arrowhead) in an early juvenile, scale bar = 1 mm. At the x axis, the numbers of the vertebral bodies, hemal spines (HS), neural spines (NS), and urostyle (Ur) are indicated.

Along the vertebral column, other common deformities were found with a variable degree of incidence (Figure 7). The most commonly deformed, compressed, and/or fused elements were those located at the end of the vertebral axis (vertebral bodies 37–40). The 39 and 40 vertebral bodies were the most abundantly deformed, with 37% and 38% of the specimens showing a deformed, compressed or fused vertebra, and vertebral fusion being the most common type of deformity (28% and 29% of specimens, respectively). Their neural and hemal spines were also the most commonly deformed (with an incidence ranging 33–37%). Vertebra 23–25 also showed an appreciable incidence of deformity, although low (ranging in an incidence of 1–3%).

**Figure 8:**
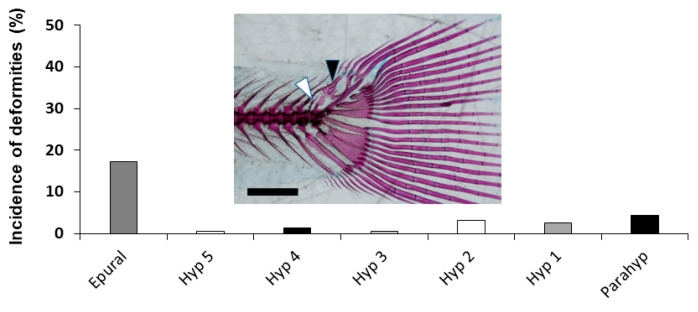
Skeletal deformities and incidence in the caudal structures of tench (*Tinca tinca*) during larval development when reared in natural ponds through double-staining analysis. Embedded image shows a caudal fin complex with deformed epural and modified neural spine (black arrowhead) and additional neural spine in the preural vertebrae 2 (white arrowhead). Skeletal structures: *Epural*, epural; *Hyp 1-5*, hypurals 1-5; and *Parahyp*, parahypural. Scale bar = 1 mm.

The other region with a high incidence of skeletal deformities was the caudal fin complex (Figure 8). All elements showed deformities, from the lowest incidence at the hypural 3 and 5 (0.64% in both) to the highest one of the epural (17.20%).

### 3.3. Juvenile Skeletal Deformities

**Figure 9:**
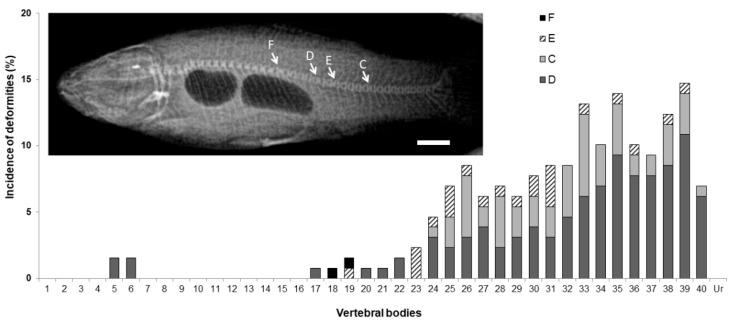
Skeletal deformities and their incidence in the vertebral column of tench (*Tinca tinca*) juveniles. Embedded image shows an example of a specimen showing the 4 different types of deformity found: fusion (F), elongation (E), compression (C), or any other kind of vertebral shape deformity (D). Nomenclature adapted from [19]. Scale bar = 1cm.

To explore the incidence of skeletal deformities in tench juveniles, 274 specimens were analyzed through X-ray images (Figure 9). Specimens smaller than 9 g of body wet weight were not analyzed since their skeleton had a low mineral density, not allowing to get clear images. The 52.2% of the X-rayed fish had at least one vertebral deformity. The identified vertebral anomalies were compression, fusion, elongation and vertebral shape deformity, all of them being mainly located in the caudal region. General deformity was most frequently located at vertebra 39 (10.85% of the individuals), vertebral compression at vertebra 33 (6.20%) and vertebral elongation at vertebra 31 (3.10%). Vertebral fusion was only registered in a low percentage of individuals (0.78%) and exclusively at vertebras 18 and 19.

## 4. Discussion

Because fish are one of the most evolved and diverse taxonomic groups, showing a wide range of phenotypes/morphologies, the study of the ontogenetic (skeletal) development for each species is required [16]. Indeed, determining the types and incidences of skeletal deformities present in reared tench is crucial to define research priorities for its aquaculture improvement and sustainability. In this sense, skeletal development during larval and juvenile stages deeply determines fish quality. Thus, to overcome this problem a better and detailed knowledge of fish species-specific skeletogenesis is required and might certainly help to characterize their etiology [12]. Furthermore, defining and implementing regular monitoring procedures for early-detection systems and quality indicators might also warrant a continuous optimization of the rearing protocols and systems used [20]. In this case, a detailed description of the sequence by which the different structures of the tench skeleton are formed and the identification and quantification of different types of deformities was herein conducted for the first time using specific methodologies.

Previous studies have already speculated whether skeletal deformities are an important bottleneck in tench aquaculture, but none of them explored this topic with specific techniques such as acid-free double-staining and/or X-rays, just being based on basic observations of the general external morphology [13,21,22,23,24,25,26]. The development of early bony ossification in fish species through an acid-free double-staining technique, reduces inaccuracies of descriptions on skeletal development due to the decalcification of small structures undergoing ossification when using an acid staining protocol [16]. Indeed, the acid-free double staining allows an early detection of altered skeletogenesis and thus, it represents an important decision-making tool for fish-farm managers. On the other hand, X-ray analysis permits a quick and clear diagnosis of skeletal deformities in fish juveniles/adults, without the need of sacrificing the specimens (allowing a follow-up until market size), revealing critical skeletal deformities such as vertebral compression and/or fusion, that escape from the visual inspection on the external morphology performed by aquaculture operators [27].

### 4.1. Tench Skeletogenesis along Larval Development

As in many other teleost fish species such as zebrafish (*Danio rerio*), gilthead seabream (*Sparus aurata*), or Senegalese sole (*Solea senegalensis*), the first skeletal elements to be formed in tench are those located in the cranial region, related to feeding (Meckel’s cartilage, ethmoid, and hyomandibular) or respiratory purposes (ceratobranchials and cleithrum) [16,17,28]. Their ossification occurred quite fast, as most of them already showed an advanced ossification at 6–12 mm of SL. In tench, similarly to other fish species, the development of the axial skeleton was sequential and bidirectional, from Weberian 3–4 vertebrae to the head and urostyle, similarly to zebrafish and gilthead seabream [18,29]. The vertebral column, including vertebral centra, neural, and haemal arches and spines, the parapophyses, and the ventral ribs are formed by intramembranous ossification. The unique exception are the arches and spines of the last centra (35–40), which undergo endochondral ossification. In tench larvae, the first ossified vertebra was identified in larvae ranging 6–12 mm of SL. The Weberian (1–4), the prehemal vertebrae (5–23), caudal (24–38), and preural vertebrae (39–40) suffered a very rapid ossification process, being fully mineralized in larvae with 12–17 mm of SL. Indeed, the last elements to be ossified in the axial skeleton were the ones located at the dorsal, pectoral, pelvic and anal fins, evidencing that an intense swimming activity in early tench specimens for feeding onto live preys might be required. Finally, regarding the caudal fin complex, its formation started with endochondral ossification of hypurals at 4–6 mm of SL, being almost completely ossified at larvae with 12–17 mm of SL, with rays being fully ossified at 17–26 mm of SL. Although there were some clear differences on the timing of the onset of ossification, the skeletal structures of tench followed similar processes of ossification (endochondral and intramembranous) previously described in other fish species [16,17,28,29,30,31,32].

### 4.2. Incidence of Skeletal Deformities in Tench Larvae and Juveniles

A large set of abiotic and biotic factors have been accounted for the appearance of skeletal deformities in fish (reviewed in [12]). In tench, different studies reported the appearance of deformities by visual screening of the external morphology during the last decade [13,21,22,23,24,25,26]. However, none of the abovementioned studies performed in tench determined the incidence of skeletal deformities with specific and accurate methodologies. Thus, the implementation of these methodologies allows one to deepen the etiologies of the particular skeletal deformities found in tench and refine rearing protocols and systems.

Specific techniques for skeletal analysis have been reported to be more accurate than visual screening to quantify the real problem of abnormal skeletogenesis in fish species. In this sense, while the 88% of the Senegalese sole population visually screened were categorized as normal, radiographic screening confirmed that 72% of these normal fish displayed abnormalities [27]. A great variability of visible deformities and their incidence have been reported, from 0 to 96.4% [13]. Here, using an acid-free double-staining protocol, 54.1% of deformed larvae/early juveniles was found when reared in a natural pond system. In a second cohort of 1-year-old tench juveniles, 52% of specimens was deformed when a X-ray analysis was performed. Those results pointed out that a considerable incidence of skeletal deformities might hamper the aquaculture of tench, similarly to other fish species, and within the range previously reported by Wolnicki and collaborators [13]. Among the main causes of skeletal deformities, nutrition, rearing system, and water temperature have been particularly reported in tench [13,24,25,26,33].

Starter diets have been found to induce a high incidence of external body deformities (77.9–96.4%) [13], probably due to an imbalanced content of some key nutrients. In this regard, Kasprzak and collaborators [34] showed a better skeletal development of crucian carp juveniles when fed natural feed (*Chironomidae sp.* larvae) rather than two popular commercial diets, suggesting that basic commercial diets might be inadequate for the intensive rearing of crucian carp juveniles. Although some attempts have been done to develop a species-specific starter diet for tench [22,23,25,33], there is still room for improvements in order to get good results as with natural feed. Water-rearing temperature during fish early development is another quite known factor inducing abnormal skeletogenesis [12]. Nevertheless, the authors of [24] reported that water temperature only modified the effects of the major dietary factor, with 90% of deformed fish when they were reared at 26 °C. When comparing rearing systems (extensive, semi-intensive, and intensive), different incidences of deformity (19.42%, 27.51%, and 27.18%, respectively), among other biological parameters, were found in tench [26]. Although the same authors attributed such differences to nutritional deficiencies associated with artificial feeding regime, the differences in abiotic rearing conditions should not be neglected. Fish reared under extensive systems (e.g., natural ponds and/or mesocosms) are known to present lesser incidence of deformities than intensive systems due to a less stressful environment, lower competition for food, and larger space availability [35,36]. Moreover, differences in the percentage of severe deformities might be also related to the higher survival ability of those specimens under intensive conditions, where there is a total lack of potential predators and high abundance and frequency of feed supply to warrant high survival rates.

Regarding the incidence and types of deformities, the abundance of severe deformities might be in accordance with their influence on the fish survival or due to the less stressful environment, lower competition for food and/or larger space availability, as previously commented. The absence or low (<5%) incidence of jaw and opercular deformities at larval and juvenile stages might be related to better rearing conditions or the compromised viability of these specimens showing these types of deformities, being less efficient to catch preys, more prone to suffer pathogen infections, or unable to cope with the higher requirements of ventilation under hypoxic conditions [37]. Indeed, within the juvenile population examined through X-ray analysis, none of them showed jaw, opercular, or pectoral fin deformities. The lack of opercular deformities might be related to the fact that in natural ponds, fish might be exposed to periodic events of oxygen deficiency since they are in stagnant waters with muddy bottom and abundant vegetation [21]. Such conditions might act as a natural selection process on those specimens showing this type of deformity which might be unable to get enough oxygen upon temporal oxygen deficiency and die [38]. Similarly, specimens showing total or partial absence of pectoral fins (approximately 9%) were found with the acid-free double-staining protocol at larval stage, but none of the 1-year-old individuals showed these types of deformity. The cause of the pectoral fin absence in tench early juveniles reared in natural ponds is obscure. One plausible hypothesis might be this absence being a result of traumatic injury (e.g., fin loss due to a predator’s bite) and/or due to a total lack of vitamin A on the larval diets, particularly due to a down-regulation of the *hoxd-9* gene, as previously described for the absence of pelvic fins in European sea bass (*Dicentrarchus labrax*) [39].

Skeletal deformities most commonly found in both developmental stages analyzed through double-staining and X-ray analyses were vertebral deformities, with the majority of them being located in the caudal region (between 10% and 40% of the specimens showed a deformed vertebra commonly located at vertebrae 39 or 40). This type of deformity and its caudal location are common features of different farmed fish species such as gilthead seabream [40], Atlantic salmon (*Salmo salar*) [19], European sea bass [39,41], Senegalese sole [16,42] or the golden pompano (*Trachinotus* ovatus) [43]. The origin of this deformity seems to be multifactorial since nutritional imbalances, water flow, and rearing temperature, among other factors, are known to cause this abnormal development on the axial and caudal fin skeleton [16,40,42,44,45,46,47,48,49]. This high incidence in the caudal vertebra, particularly on the preural vertebrae, might be due to being the last ones to be ossified (12–17 mm of SL) and has been previously ascribed to suboptimal nutritional and rearing conditions during its formation in other fish species [16]. Such findings clearly evidenced how important it is to determine the developmental timing of skeletal structures for each fish species in order to improve rearing protocols.

Finally, lower incidence (<20%) of skeletal deformities were found in the structures from the caudal fin complex in early stages of development. A diverse set and higher incidence of deformities in the caudal fin complex of other fish species have been reported [16,40,42,43,50,51]. Nevertheless, because those deformities did not affect the locomotive performance of the fish and/or their viability, they are generally considered as non-severe deformities.

## 5. Conclusions

The application of specific techniques (double-staining and X-ray analyses) to study the tench’s skeleton might help to identify and improve suboptimal conditions during its rearing process and to perform an accurate assessment of the skeletal deformity incidence under different rearing systems and protocols, which are otherwise difficult when a simple visual inspection of the external morphology is performed. 

Skeletal formation in tench progressed very rapidly, with first elements mineralizing at 4 mm (5 dpf) and the skeleton being almost completed at 26 mm (85 dpf). Furthermore, a considerable incidence of deformities (54.1% and 52.2%) was determined using both analytical approaches in tench when reared in natural ponds. Unfortunately, due to the multifactorial cause of skeletal deformities, those described herein cannot be ascribed to a particular factor (e.g., low egg quality, suboptimal feeding in quantity and/or quality, suboptimal water temperature, genetic inbreeding, etc.). Nevertheless, taking into account the size of fish sampled and the types and incidences of the deformities found here, at least two sampling sizes for regular monitoring of the skeletal development in tench hatcheries with an acid-free double-staining technique are recommended in the present study: at 14 mm of SL, to determine the incidence of severe deformities located at the jaw, opercula and/or the pectoral fin; and at 26 mm of SL to determine the incidence and typology of skeletal deformities in the vertebral column. Furthermore, a third sampling size, at juvenile stage (7.7 to 13.8 cm of SL) and applying a X-ray analysis, might be also recommended to assess the batch of fishes reared until commercial size.

## Data Availability

The data presented in this study are available on request from the corresponding author.

## Supplementary Materials

The following are available online at https://www.mdpi.com/2076-2615/11/3/621/s1. Figure S1: Standard-length growth curve of tench (*Tinca tinca*) during larval development in natural ponds.
