# Supplementary material for: Skeletal Development and Deformities in Tench (Tinca tinca): From Basic knowledge to Regular Monitoring Procedure
Source: Animals (Basel). 2021 Feb 26;11(3):621. doi: 10.3390/ani11030621 (PMC7996733; doi:10.3390/ani11030621)
Supplement: Supplementary file 1 [file animals-11-00621-s001.pdf]

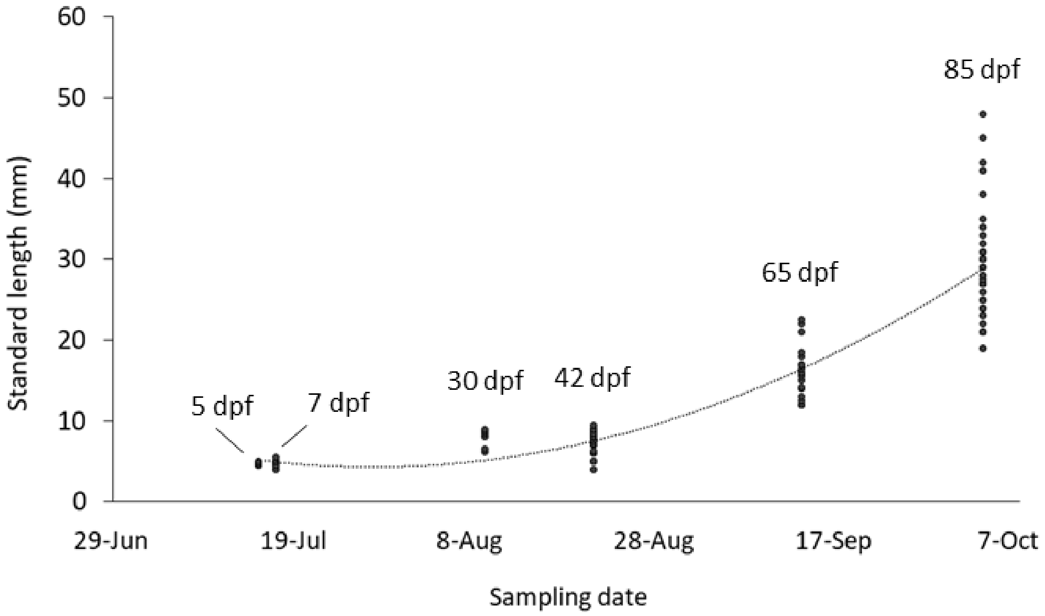

Figure S1. Growth of tench (*Tinca tinca*) in standard length (mm) when reared in natural ponds. Larval age is shown above each sampling point and corresponding to 5, 7, 30, 42, 65 and 85 days post-fertilization (dpf) Data distribution follows an exponential curve (dotted line; $y = 0e^{0.025x}$ with $R^2 = 0.8793$), characteristic of the fish growth during larval development.
